# Preparation and Characterization of Loperamide-Loaded Dynasan 114 Solid Lipid Nanoparticles for Increased Oral Absorption In the Treatment of Diarrhea

**DOI:** 10.3389/fphar.2016.00332

**Published:** 2016-09-21

**Authors:** Lili Wei, Yunfang Yang, Kun Shi, Jun Wu, Wei Zhao, Jingxin Mo

**Affiliations:** ^1^Department of Pharmacy, Affiliated Hospital of Guilin Medical UniversityGuilin, China; ^2^Department of Cardiology, The Second Affiliated Hospital of Guilin Medical UniversityGuilin, China; ^3^Department of Gynecology, Guangzhou Women and Children’s Medical CentreGuangzhou, China; ^4^School of Engineering, Sun Yat-sen UniversityGuangzhou, China; ^5^Department of Histology and Embryology, Zhongshan School of Medicine, Sun Yat-sen UniversityGuangzhou, China; ^6^Key Laboratory for Stem Cells and Tissue Engineering, Ministry of Education, Sun Yat-sen UniversityGuangzhou, China

**Keywords:** poorly water-soluble drugs, solid lipid nanoparticles, sustained release, oral delivery formulation, loperamide

## Abstract

The aim of the project was to assemble two optimum solid lipid nanoparticle (SLN) formulations for oral delivery of loperamide (LPM) to treat different types of diarrhea, and to evaluate their release profiles *in vitro* and pharmacokinetic properties *in vivo*. In this work, glyceryl trimyristate (Dynasan 114) nanoparticles containing the drug LPM and sodium cholate as a stabilizer were prepared using a modified solvent evaporation technique. Two LPM-loaded SLNs, namely LPM-SLN-1 (LPM-SLN with a high ratio rate of lipid to drug) and LPM-SLN-2 (LPM-SLN with a low ratio rate of lipid to drug), were prepared by the solvent evaporation method. A change in the lipid concentration affects the characteristics of LPM-SLNs. The average sizes of the LPM-SLNs were 303 ± 18 nm and 519 ± 36 nm, separately, as analyzed by dynamic light scattering. The LPM-SLNs were found to be round with a smooth surface, as observed using a transmission electron microscope and a scanning electron microscope. The average encapsulation efficiencies were 87 ± 3.78% w/w and 84 ± 5.17%, accordingly. In the *in vitro* release experiments, LPM-SLNs showed a continuous release profile of LPM without any burst release. The oral bioavailability of LPM-SLNs was analyzed using Wistar rats. The relative bioavailabilities of LPM-SLNs were 227 and 153%, respectively, as compared that of the LPM tablet. There was no difference in the T_max_ between LPM-SLN-2 and the LPM tablet. In conclusion, LPM-SLN-1 significantly improved the oral bioavailability of LPM, while LPM-SLN-2 having the same swift action as the LPM tablet. These results demonstrate the potential of LPM-SLNs in the oral delivery of LPM to treat different types of diarrhea.

## Introduction

Loperamide (LPM) is an orally administered antidiarrheal agent indicated for the short-term treatment of acute and chronic diarrhea in adults (Natalja et al., 2013). LPM is available in a range of orally administered formulations, including tablets, capsules, a combination chewable tablet, and an orodispersible formulation (Trottet et al., 2004). However, LPM has limitations in oral administration owing to its extreme bitter taste. Moreover, LPM is a poorly water-soluble drug. Its slow dissolution rate in the intestinal tract and its significant first-pass effect largely restrict its clinical use (Trottet et al., 2004). To overcome these challenges, Ueda and Kreuter (1997) had successfully loaded LPM into Poly(L-lactide) nanoparticles to increase its solubility and to improve its release profile *in vitro* (Ueda and Kreuter, 1997). However, there are few reports on the formulation of LPM solid lipid nanoparticles (SLNs), which could be a possible way to overcome the limitations in the oral absorption of LPM.

Delivering drugs to their site of therapeutic action is one of the main challenges faced by the pharmaceutical industry (Parveen et al., 2012). This can be due to a range of factors, such as the chemical properties of the drug, biological processes, or patient factors, such as age or disease state (Mehnert and Mader, 2001; Perrie et al., 2012). An exciting area of research aiming to overcome some of these limitations is nanotechnology. In particular, the pharmaceutical industry is keenly interested in research into nanoencapsulation, which involves the formation of drug-loaded particles with a diameter of 1-1000 nm (Pinto Reis et al., 2006). This technology has many potential benefits, including improved drug bioavailability and solubility, increased plasma half-life, selective targeting, reduced toxicity, increased stability, and provision for controlled drug release (Budhian et al., 2007; Luo et al., 2011; Danhier et al., 2012; Parveen et al., 2012; Wang et al., 2012a,b).

One important area of nanoencapsulation is the development SLNs. SLNs encompass a broad range of colloidal systems, which can include micro emulsions, liposomes, and polymeric nanoparticles (Schwarz and Mehnert, 1997; Mehnert and Mader, 2001; Luo et al., 2011). The nanoparticles consist of a matrix made up of a solid lipid shell (solid at room and body temperature; Luo et al., 2011; Fathi et al., 2012) that is stabilized by emulsifiers and contains a loaded drug cargo (Luo et al., 2011; Parveen et al., 2012; Mo et al., 2015). SLNs combine the advantages of various colloidal carrier systems (Harde et al., 2011; Zhang et al., 2012) by incorporating the advantages of the solid matrix of polymeric nanoparticles with the advantages of micro emulsions and liposomes in having low biological toxicity (Casadei et al., 2006; Harde et al., 2011). Glyceryl trimyristate (Dynasan 114) is a triglyceride that has been the component of several novel SLN formulations (Thava Seelan and Leggat, 2003; Leggat, 2005). Dynasan 114 SLNs have demonstrated the ability to be degraded within 60-120 min in the simulated gastrointestinal tract, when combined with a suitable surfactant such as sodium cholate (Thava Seelan and Leggat, 2003; Leggat, 2005). It has also been shown to be non-toxic for human use and is officially recognized as a pharmaceutical excipient. It is used as a lubricant in tablets and as a seeding/crystallization agent to improve the solidification process in suppositories, vaginal ovules, and cosmetic sticks. The combined low toxicity and degradative properties suggest a potential for drug-loaded Dynasan 114 SLN to be used in orodispersible formulations (Thava Seelan and Leggat, 2003; Leggat, 2005; Wilder-Smith, 2008). Nanoencapsulation of a drug in Dynasan 114 SLN can potentially mask the unpleasant taste of the drug, eliminate the gritty sensation within the mouth offered by micron-sized particles (<3 μm), while allowing for the rapid release of the incorporated drug upon Dynasan 114 degradation in the gastrointestinal tract.

The aim for the study is to assemble stable LPM-loaded SLNs and further explore the possibility of using these novel formulations to cure different kinds of diarrhea. LPM-loaded Dynasan 114 SLNs were prepared using the solvent evaporation method, with modifications (Mehnert and Mader, 2001; Wang et al., 2012b). The formulation was evaluated for its mean diameter (MD), polydispersity index (PDI), zeta potential (ZP), and drug loading (DL) and entrapment efficiency (EE). The effects of freeze drying on the stability of the formulation were also investigated. Then, the LPM release from SLN was investigated *in vitro*, and its oral bioavailability was analyzed *in vivo*.

## Materials and Methods

### Materials

Chemicals used for the synthesis of nanoparticles were loperamide (>99%, Sigma Aldrich, St Louis, MO, USA), sodium cholate hydrate (>99%, Sigma), glyceryl trimyristate (Dynasan 114; >99%, Sigma), and dichloromethane (>99.5%, Sigma). Purified water (MilliQ, Millipore, USA) was used throughout and all other chemicals were of analytical grade. All experiments were conducted at room temperature (25°C). Loperamide tablets were purchased from Xian Janssen Pharmaceutical Ltd, Xi’an, China.

### Preparation of Loperamide-Loaded Solid Lipid Nanoparticles

The loperamide-loaded SLNs (LPM-SLNs) were prepared by a solvent evaporation technique, with modifications (Mehnert and Mader, 2001; Wang et al., 2012b). The non-aqueous phase consisted of 250 mg of Dynasan 114 and 5 mg or 15 mg of loperamide (50:1 or 16.7:1 w/w) dissolved in 1.5 ml of dichloromethane. The aqueous phase consisted of 50 mg of sodium cholate (added to give a weight ratio of 1:5 to Dynasan 114) dissolved in 30 ml of purified water. The non-aqueous phase was added dropwise into the aqueous phase under rapid stirring at 500 rpm for homogenous dispersion (Magnetic Stirrer CS76083V, Industrial Equipment and Control Pty LTD, Australia). After 1 min of stirring, the homogenous dispersion was sonicated (Vibra cell Model VCX130, Sonics and Materials Inc, USA) at 80% amplitude for 5 min. The dispersion was transferred to a rotary evaporator in a 50-ml round bottom flask and the organic solvent was allowed to evaporate over 30 min at 80 rpm at 25°C (Rotavapor R-210, Buchi Labortechnik, Switzerland). The dispersion was collected and analyzed, or freeze dried for further analysis. The composition of each of the different formulations is summarized in **Table 1** and referenced by identification name.

**Table 1:** Composition of loperamide loaded solid lipid nanoparticles.

ID Name	Loperamide HCl (mg)	Sodium cholate (mg)	Dynasan 114 (mg)	Ratio
LPM-SLN-1	5	50	250	1: 10: 50
LPM-SLN-2	15	50	250	1: 3.3: 16.7

### Mean Diameter, Polydispersity Index, and Zeta Potential of SLN Samples

The particle size and PDI were determined by dynamic light scattering (DLS; Zetasizer Nano ZS, Malvern Instruments Ltd, Worcestershire, UK). Particle size, PDI, and ZP measurements of the SLN dispersion were performed immediately after manufacturing, and again after the dispersion had been freeze dried and reconstituted in purified water. In all cases, the dispersion was measured immediately after it was allowed to equilibrate for 2 min in the equipment.

### Transmission Electron Microscope (TEM) and Scanning Electron Microscope (SEM) Analysis

The morphology of LPM-SLN formulations was studied using transmission electron microscopy (JEM-1200EX, JEOL; Mo et al., 2016). One drop of LPM-SLNs was diluted 50-fold with pure water before placing onto a 400-mesh carbon film, copper grid, and then negatively stained with 1% phosphotungstic acid. The sample was air-dried before TEM inspection (Mo et al., 2016).

The surface morphology of LPM-SLN formulations was observed by scanning electron microscopy (1555VP FESEM, Zeiss, Jena, Germany; Mo et al., 2015). Before observation, the lyophilized samples were fixed on a double adhesive carbon tape, which was stuck on aluminum stubs and then coated with gold under an argon atmosphere. The samples were visualized by SEM with an accelerating voltage of 8–20 kV (Mo et al., 2015).

### HPLC Analysis of LPM

The mass analysis of LPM was performed by reverse-phase HPLC. The HPLC system consisted of a model LC-10AT pump (Shimadzu, Kyoto, Japan) and a model SPD-10A UV detector (Shimadzu; Mo et al., 2011). The stationery phase was Diamonsil C_18_ (200 × 4.6 mm, 5 μm; Dikma, USA). The injection volume was 20 μl; a mixture of 0.1 mol/l ammonium carbonate and acetonitrile (90:10, v/v) was used as a mobile phase with a flow rate of 1.0 ml/min. The UV detection wavelength was 264 nm; the column temperature was 30°C.

### Entrapment Efficiency and Drug Loading

The EE of LPM-SLNs was calculated by determining the amount of free drug obtained after ultrafiltration (Mo et al., 2015). One milliliter LPM-loaded SLN colloidal solution was ultrafiltered for 10 min at 4000 rpm. The ultrafiltrate containing the unencapsulated drug was analyzed using HPLC. The total LPM in LPM-SLNs was determined by breaking up the LPM-SLNs as described previously, with minor modifications (Mo et al., 2014b). Aliquots of 1 ml LPM-SLN dispersions were vortexed with 2.5 ml of methanol for 5 min, followed by filtration through 0.45 μm membrane filters. The filtrate was transferred into glass autosampler vials and a 20-μl aliquot sample was injected into HPLC, as mentioned above, for analysis. The DL was the weight of the encapsulated drug divided by the weight of the lipid (w/w). The EE and the DL were calculated by the following equations:

$$\begin{array}{c} \mathrm{EE}\;(\%)=\frac{W_{Total}-W_{Free}}{W_{Total}}\times 100\%\\ \mathrm{Drug}\;\mathrm{loading}\;(\%)=\frac{W_{Total}-W_{Free}}{W_{Lipid}}\times 100\% \end{array}$$

### Freeze Drying and Reconstitution

LPM-SLN dispersions were frozen at -40°C for 24 h (Medical freezer, Model MDF135, Sanyo Ltd, Japan). The frozen samples were then freeze dried (VirTis Freezemobile 35 EL, SP Scientific) over 48 h to yield a powdery sample. To determine whether the freeze-dried powder could be reconstituted into SLNs, 30 mg of the powder was resuspended in 3 ml of purified water to yield the same concentration of SLNs (1% w/v) as was present in the initial dispersion prior to freeze drying. To aid the dispersion of the SLN, the resuspended sample was either vortexed for 5 min at 2400 rpm (Heidolph Reax Top, John Morris Scientific Pty Ltd, Germany) or sonicated for 2 min at 80% amplitude (Vibra cell Model VCX130).

### *In vitro* Release Studies

The release rates of LPM from LPM-SLN-1, LPM-SLN-2, and LPM tablets were investigated using dialysis bags at pH 1.2 and pH 6.8. LPM-SLN-1, LPM-SLN-2, and LPM tablets equivalent to 10 mg of LPM were placed into a dialysis bag (Spectrum Laboratories, Inc., Rancho Dominguez, CA, USA; molecular weight cut off, 3500 Da) containing 1000 ml PBS (pH 1.2 or 6.8) with 1% Tween 80, at 37.5°C. At different time points, the release medium in which the dialysis bag was immersed was sampled (5 ml) for analysis by the above mentioned HPLC method.

### *In vivo* Pharmacokinetics

All animal experiments were carried out following the Principles of Laboratory Animal Care (People’s Republic of China; Mo et al., 2014a), and the protocols for the animal studies were approved by the Department of Laboratory Animal Research at Guilin Medical University (License No. YXLL-2015-039). Male Wistar rats (7-8 weeks old) weighing 200–250 g were obtained from Animal experimental center of Guilin Medical University and housed at a temperature of 25 ± 2°C. The rats were divided randomly into three groups containing six animals each and fasted overnight before the experiment, but had free access to water. Fasting minimizes the influence of food on intestinal absorption of LPM and its formulations.

The rats were weighed before the administration of LPM-SLNs (5 mg/ml) or an equivalent dose of the LPM tablet by gavage at a dose of 5 μl/g of body weight. Blood samples were collected as previously reported (Mo et al., 2014a). Briefly, 0.5 ml blood was sampled from each animal, via the suborbital vein using capillary tube method at 0, 0.17, 0.33, 0.5, 1, 2, 3, 4, 5, 6, 8, 10, 12, and 16 h after administration. All blood samples were immediately centrifuged at 5000 rpm for 10 min to separate the plasma. The plasma obtained was stored at -80°C until analysis.

The concentrations of LPM in rat plasma were determined by HPLC as mentioned above. Samples were prepared as following: 0.4 ml plasma was mixed with 0.2 ml 0.3 M NaOH and vigorously vortexed for 1 min. Then, 2 ml of ethyl acetate was added and the mixture was vortexed for 5 min. The organic layers were collected after centrifugation at 5000 rpm for 5 min, and evaporated to dryness. The residue was dissolved in 0.2 ml of the mobile phase by vortexing for 5 min and 20 μl was injected into the HPLC.

The major pharmacokinetic parameters were determined by DAS 2.0 (Mathematical Pharmacology Professional Committee of China, Shanghai, China). The values of maximum concentration (*C*_max_) and time of maximum concentration (*T*_max_) were read directly from the concentration–time curves. Plasma concentration vs. time data were fitted into a two-compartment model to obtain estimates of the area under the plasma concentration–time curve during the period of observation (AUC_0-t_). The relative bioavailability of the SLN formulations was calculated by the following formula:

$$\mathrm{Fr}(\%)=\frac{AUC_{T}D_{R}}{AUC_{R}D_{T}}\times 100\%$$

where, *Fr* was the relative bioavailability, *AUC* was the area under the plasma concentration, *D* was the dose administrated, *T* was the test formulation (oral administration of LPM-loaded SLNs formulations), *R* was the reference formulation (oral administration of LPM tablet; Mo et al., 2014a).

### Statistical Analysis

All batches were produced in triplicates except when mentioned otherwise. All values are expressed as mean ± SD. Statistical analysis of the mean values between batches was performed using the Student’s *t*-test (Excel 2010, Microsoft, Redmond, WA, USA). The differences were considered significant when *p* < 0.05.

## Results and Discussion

### Physiochemical Characteristics of LPM-SLNs

**FIGURE 1:**
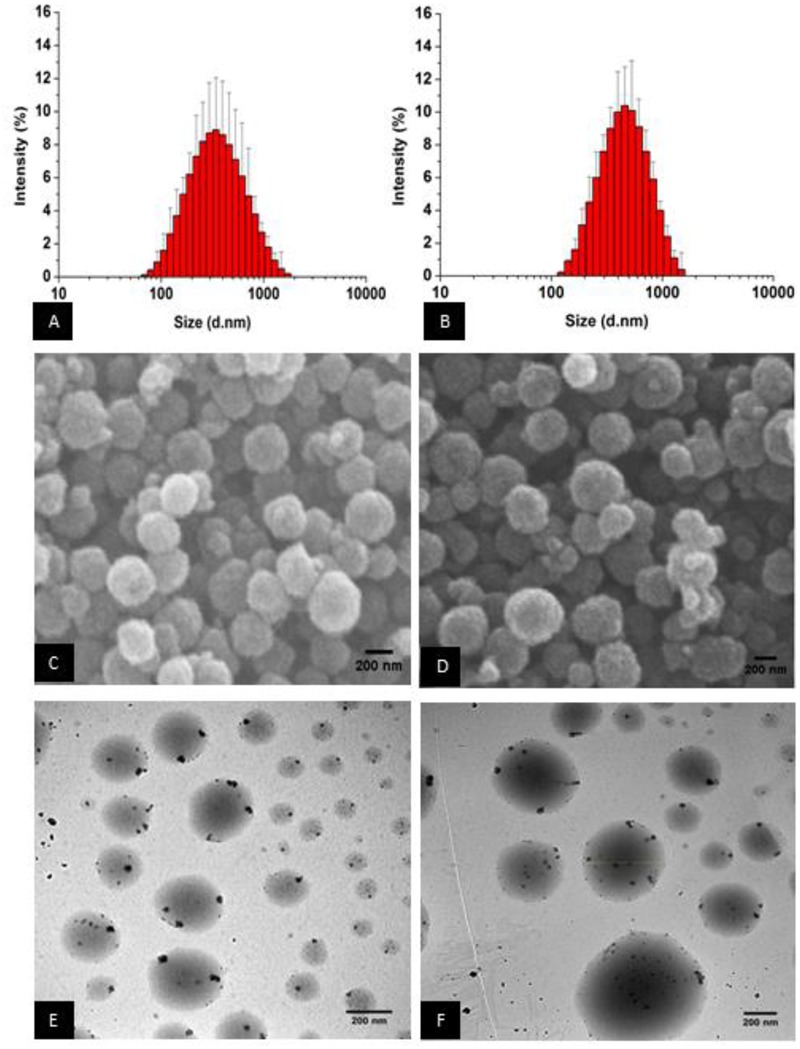
**Loperamide-loaded Dynasan 114 solid lipid nanoparticles [LPM (loperamide)-SLN (solid lipid nanoparticle)]. (A)** *Z*-average size of LPM-SLN-1, **(B)** *Z*-average size of LPM-SLN-2, **(C)** SEM image of LPM-SLN-1, **(D)** SEM (scanning electron microscope) image of LPM-SLN-2, **(E)** TEM (transmission electron microscope) image of LPM-SLN-1, **(F)** TEM image of LPM-SLN-2; scale bar 200 nm.

The DLS measurements for LPM-SLN-1 showed a mean size of 303 ± 18 nm, with a PDI of 0.19 ± 0.01. A histogram of the size distribution showed a peak at 342 nm and a normal distribution with size range of 68-1484 nm (**Figure 1A**). ZP measurements suggested that LPM-SLN-1 possessed a high negative surface charge of -47.33 ± 4.29 mV.

The DLS measurements for LPM-SLN-2 showed a mean size of 519 ± 36 nm, with a PDI of 0.18 ± 0.01 and ZP of -54.63 ± 8.12 mV. The size distribution histogram showed a slightly broader continuous distribution with a peak at 458 nm and a slight positive skew between 122 and 1484 nm (**Figure 1B**). The particle sizes of three batches of LPM-SLN-1 and LPM-SLN-2 were 296.65 nm, 323.75 nm, and 289.6 nm; and 526.84 nm, 550.69 nm, and 479.47 nm, respectively. The MD of the two formulations was significantly different (*p* = 0.00295). However, LPM-SLN-2 did not show a statistically significant difference in ZP (*p* > 0.05) when compared to LPM-SLN-1. Interestingly, there was no increase in the PDI (*p* > 0.05), confirming that an increase in the drug load in LPM-SLN-2 did not widen the nanoparticle size distribution, as compared to LPM-SLN-1.

### Morphology of LPM-SLN Formulations

**Figures 1C,D** show the images of surface morphology of LPM-SLNs. The results showed that the particles were spherical with a narrow size distribution, and had smooth surfaces. No crystals of the drug or aggregation of nanoparticles was found in the sample. The morphology of LPM-SLNs determined by TEM is shown in **Figures 1E,F**. The TEM study showed that the particles had almost round and uniform shape and did not stick to each other.

### HPLC for Determination of Loperamide after the Production of Loperamide SLN

**FIGURE 2:**
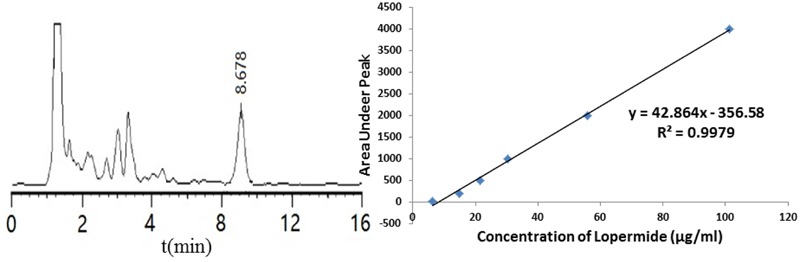
**(A) Typical chromatograms for loperamide HCl. (B)** Calibration curve obtained for loperamide HCl in the range of 6-100 μg/ml.

A calibration curve of absorbance versus concentration was plotted using standard solutions of loperamide HCl using HPLC. As shown in **Figure 2**, a linear regression analysis of the data produced a line with an *R*^2^ value of 0.9979 and the equation:y = 42.864x - 356.58. The retention time for LPM was 8.7 min.

### Drug Loading and Entrapment Efficiency

Drug loading and entrapment efficiency were calculated using the equations described in the Methods section. A DL of 1.57 ± 0.31% w/w and 4.25 ± 0.38% w/w and an EE of 87 ± 3.78% w/w and 84 ± 5.17% w/w were obtained for LPM-SLN-1 and LPM-SLN-2, respectively.

### Freeze Drying

**FIGURE 3:**
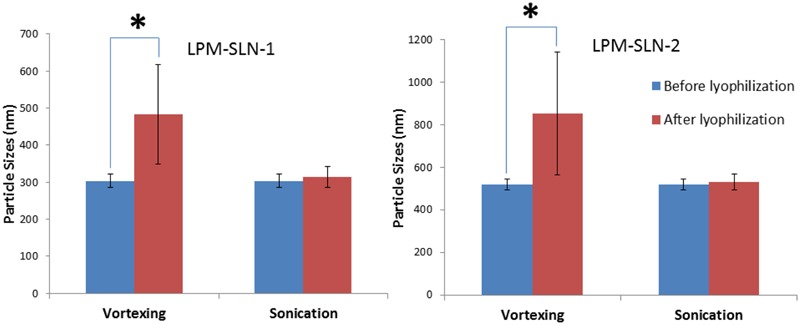
**Particle sizes of LPM-SLN formulations after lyophilization and reconstitution by vortexing or sonication.** ^∗^*p* < 0.05.

The LPM-SLN samples were freeze dried to determine whether the SLNs were amendable to freeze drying. The freeze-dried samples were reconstituted in purified water by either vortexing or sonication. As shown in **Figure 3**, vortexing resulted in the reconstituted samples exhibiting a large MD of 483 ± 135 nm for LPM-SLN-1 and 853 ± 289 nm for LPM-SLN-2; a PDI of 0.44 ± 0.08 and a ZP of -29.6 ± 0.53 mV for LPM-SLN-1; a PDI of 0.42 ± 0.11 and a ZP of -25.1 ± 2.47 mV for LPM-SLN-2. Conversely, sonication resulted in the samples having an MD of 313 ± 28 nm for LPM-SLN-1 and 531 ± 36 nm for LPM-SLN-2; a PDI of 0.13 ± 0.08 and a ZP of -49.6 ± 0.42 mV for LPM-SLN-1; a PDI of 0.22 ± 0.07 and a ZP of -38.1 ± 3.27 mV for LPM-SLN-2. The samples reconstituted via vortexing had significantly larger MD, PDI, and smaller ZP values, as compared to reconstitution via sonication (*p* < 0.05). The sonicated samples had similar MD and PDI values as the freshly prepared samples. These experiments suggest that freeze-dried LPM-SLNs were fairly well reconstituted by sonication. Lyophilization is a possible way to preserve LPM-SLNs.

### *In vitro* Release Study

**FIGURE 4:**
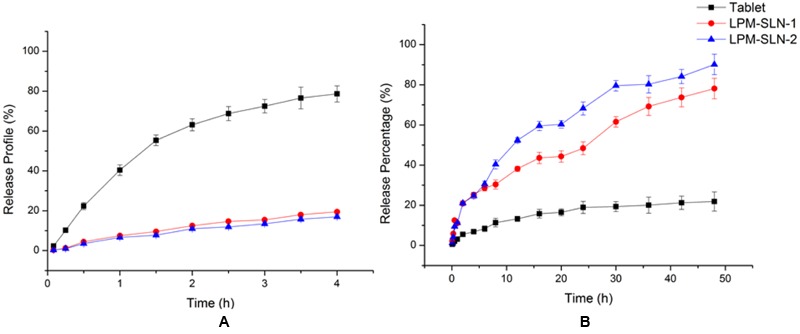
***In vitro* release profile of LPM from different vehicles at **(A)** pH 1.2 and **(B)** pH 6.8. Each value represents the mean ± S.D.** (*n* = 3).

The LPM release profiles at different pHs are shown in **Figure 4**. The release profiles of LPM-SLNs at pH 1.2 and pH 6.8 showed no burst releases of LPM at the beginning in both media, which verifies that there was little drug attached to the surface of SLNs. A continuous release profile was observed at pH 6.8. The accumulated dissolution of LPM from SLNs in PBS (pH 6.8) over 48 h was about 80%, whereas, it was less than 20% from commercial tablets (Mo et al., 2014a). However, LPM commercial tablets released more drug (up to 80%) at pH 1.2 in 4 h because of the weakly alkaline nature of LPM, while it was less than 20% in LPM-SLNs. These results indicate that LPM was encapsulated in SLNs and was protected from the strong acidic environment within the stomach. After the LPM-SLNs reach the small intestine, the intact LPM would be released from the LPM-SLNs. The results suggested that the majority of the LPM in SLNs could be taken up by intestinal cells. The release profiles of LPM-SLN-1 and 2 were similar at pH 1.2, but different at pH 6.8. The release of the drug from LPM-SLN-1 was slower than that from LPM-SLN-2.

The release profiles of the drug from LPM-SLN-1 and 2 in PBS (pH 6.8) were fitted to a Ritger–Peppas kinetics model using Origin 8.5 and the following equations were obtained: ln *R* = 0.8071 ln *t*- 0.2452 (*r* = 0.9896) for LPM-SLN-1 and ln *R* = 0.7549 ln *t* - 0.3147 (*r* = 0.9934) for LPM-SLN-2. Based on the fitting result using the Ritger–Peppas model (ln *R* = *k* ln *t* + *C*; Mo et al., 2011), the values of *k* were 0.8121 and 0.6954, respectively (0.45 < *k* < 0.85), which indicated that LPM release from SLNs was due to drug diffusion and lipid matrix corrosion. (Zhuang et al., 2010) The *k* value was close to 0.85, which indicated that matrix corrosion is the leading cause of drug release and most of the drug is located inside SLNs. A minor amount of the drug within the shell could diffuse into the medium.

### *In vivo* Pharmacokinetics

**FIGURE 5:**
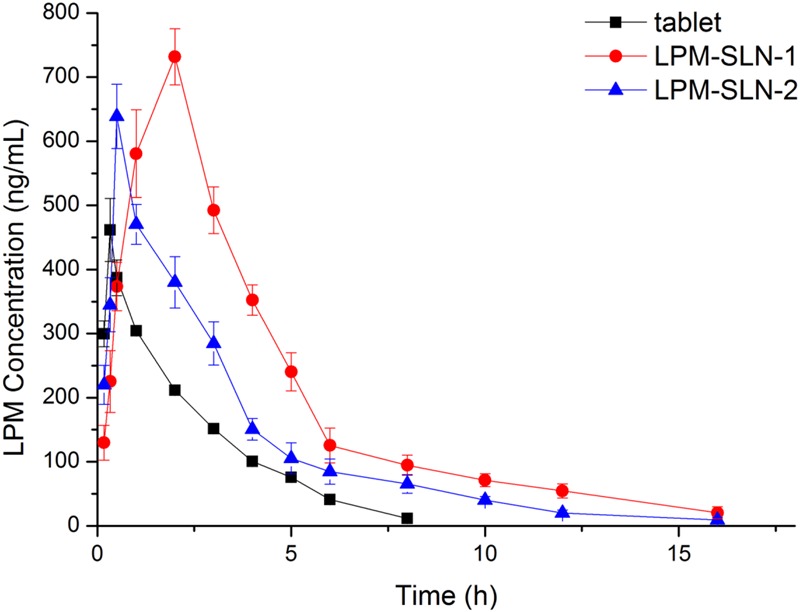
**Mean plasma LPM concentration-time curves obtained after oral administration in Wistar rats. Each value represents the mean ± S.D. (*n* = 6)**.

**Figure 5** shows the plasma concentration-time profiles of LPM after oral administration of different LPM formulations to male Wistar rats. The corresponding PK parameters are given in **Table 2**. The *T*_max_ was 0.33 h and the *C*_max_ was 461.72 ± 49.15 ng/ml when the LPM tablet was orally administrated. The *T*_max_ of LPM-SLN-1 (2 h) was about one and half hour later than that of the LPM tablet. The difference between *T*_max_ values of LPM-SLN-1 and the LPM tablet demonstrated that the absorption modes of the two formulations were different. The LPM in the tablet dissolved in the intestinal tract and was absorbed directly into the systemic circulation. Therefore, the plasma concentration of LPM quickly reached the plateau in 0.33 h. However, the drug from LPM-SLN-1 was slowly released into the gastrointestinal tract, which was verified by the *in vitro* release experiments. Interestingly, the *T*_max_ values were not significantly different between LPM tablet and LPM-SLN-2, which may be attributed to the bigger size of LPM-SLN-2, leading to an easy diffusion of the drug from the lipid matrix. The *C*_max_ values of LPM-SLN-1 and 2 were 731.87 ± 43.89 ng/ml and 638.98 ± 50.06 ng/ml, respectively, which were substantially higher than that of the LPM tablet (461.72 ± 49.15 ng/ml). At all except the first three time points, the LPM plasma concentrations in rats treated with LPM-SLNs were significantly higher than those treated with the LPM tablet. (Mo et al., 2014a) Sixteen hours after oral administration of LPM-SLNs-1 and 2, the LPM plasma concentrations were 20.47 ng/ml and 9.47 ng/ml, respectively; although, the drug concentration was 11.47 ng/ml 8 h after treatment with the LPM tablet. The AUC_0→_*_t_* after oral administration of LPM–SLN-1 and 2 were 955.35 ± 54.40 ng/ml/h and 643.43 ± 12.14 ng/ml/h, respectively, which were nearly 2.3- and 1.5-fold higher than that of the LPM tablet (421.45 ± 23.12 ng/ml/h).

**Table 2:** Pharmacokinetic parameters determined from the plasma concentration-time profiles of Wistar rats after oral administration with LPM (loperamide)-SLN (solid lipid nanoparticle)-1, LPM-SLN-2, and LPM tablet equivalent LPM dose of 5 mg/kg body weight. (*n* = 6; Mean ± SD).

	LPM tablet	LPM-SLN-1	LPM-SLN-2
	
parameters	Mean ± SD	Mean ± SD	Mean ± SD
AUC_(0-t)_ (ng/ml^∗^h)	421.45 ± 23.12	955.35 ± 54.40^∗^	643.43 ± 12.14^∗^
C_max_ (ng/ml)	461.72 ± 49.15	731.87 ± 43.89^∗^	638.98 ± 50.06
T_max_ (h)	0.33 ± 0	2 ± 0^∗^	0.5 ± 0
V_d_ (L/kg)	155.00 ± 33.62	57.02 ± 6.78^∗^	75.95 ± 5.30
CL (L/h/kg)	122.19 ± 7.37	58.39 ± 2.73^∗^	75.64 ± 2.43
t_1/2_ (h)	1.28 ± 0.45	6.73 ± 8.64^∗^	3.57 ± 1.38^∗^

*^∗^*p* < 0.05, comparison with LPM tablet.*

Since the equal amount of LPM in SLN formulations and tablet was used for *in vivo* experiment, relative bioavailability of SLN formulations is determined by dividing AUC_0-t_ of LPM SLN formulations by AUC_0-t_ of LPM tablet. Thus, Frs of LPM-SLN-1 and LPM-SLN-2 were 227 and 152%, correspondingly, which meaned more LPM in SLNs crossed intestinal barrier into systemic circle, compared to LPM tablet.

The results suggest that the systemic absorption of LPM was drastically improved by incorporation into SLNs as compared to tablets (Zhuang et al., 2010). Higher *C*_max_ and sustained release profile of LPM-SLN-1 would result in better treatment of chronic diarrhea. While LPM-SLN-2 had similar *T*_max_ as the LPM tablet, its profile makes it more suitable for treatment of acute diarrhea. Overall, the LPM-SLNs indicate an encouraging potential of increasing the oral bioavailability of water-insoluble drugs along with an ability to mask unpleasant taste.

## Discussion

The main factors contributing to the low bioavailability of LPM are its poor water-solubility, significant first pass effect in the liver, and the obstacle of the intestinal epithelial cell. Hence, we used SLNs to overcome these problems and propose the mechanism by which the effects are obtained.

First of all, SLNs are composed of different lipids whose structures are similar to the fat found in foods. The lipids could stimulate bile secretion to the small intestinal and the LPM released from SLNs was mixed with bile salts to form micelles which would improve its solubility (Zhuang et al., 2010).

Secondly, as LPM is a weakly alkaline molecule, it forms salt at a low pH (1.2), which subsequently increases its solubility. After oral administration of LPM tablets, the drug would quickly dissolve in the gastric fluid (Zhuang et al., 2010); and upon moving to the intestinal tract, the drastic increase in the pH would cause the precipitation of the drug (Zhuang et al., 2010). This transition would cause its adherence to the wall of the intestinal tract. The sustained release characteristics of SLNs could achieve a longer retention time *in vivo*.

Finally, sodium cholate was used as a surfactant in this study, which may increase the LPM absorption. Sodium cholate is one of the bile salts, and might improve the intestinal epithelial permeability by interrupting the cell membrane (Zhuang et al., 2010). Sodium cholate can attach to the *p*-glycoprotein irreversibly and inhibit the *p*-glycoprotein eﬄux pump, which would increase the attachment of LPM to the intestinal mucosa (Mo et al., 2014a).

Solid lipid nanoparticles were designed in the 1990s as an alternative to the existing drug delivery systems, such as emulsions, liposomes, and polymeric nanoparticles (Doktorovova et al., 2014). SLNs are formulated either with physiological lipids or with lipids and are have a generally regarded as safe (GRAS) status. SLNs can protect sensitive drug molecules from the external surroundings and even have controlled release profiles. However, common disadvantages of SLNs are often related with their low drug loading capacity, unpredictable gelation tendency, and so on. Drug loading capacity depends highly on the crystallinity of the lipids. In the current study, glyceryl trimyristate, which was used as the lipid, is an ester with the chemical formula C_45_H_86_O_6_. It is a long chain saturated fat. However, the encapsulated loperamide is a complex compound with three benzene rings. Thus, the high crystallinity of glyceryl trimyristate may lead to a rather low loperamide loading capacity.

Many attempts have been made to address the issue of low loading capacity. Nanostructured lipid carriers (NLC) were initially introduced (Muller et al., 2000). In these formulations, lipids of highly ordered crystalline structures are mixed with different lipids of amorphous structures, to obtain structured matrices that accommodate more drugs (Bondi and Craparo, 2010). The drug loading capacity of SLNs was increased by combining natural solid lipids with lipids of different chain lengths (Parhi and Suresh, 2012). It was noted that lipids could form matrices of scrambled structures composed of mixed crystals and mixtures of crystals, which enhanced drug incorporation as compared to a single lipid (Weber et al., 2014).

Wassim Abdelwahed (2015) prepared LPM-loaded SLNs by high shear homogenization method. A mixture of beeswax, carnauba wax, and egg lecithin was used as lipids to encapsulate LPM into the nanoparticles. This resultant drug-loaded SLN was found to have the optimal particle size for brain targeting. More importantly, the researcher revealed that using 5% monosaccharide glucose solution as cryoprotectant could better help LPM loaded-SLNs withstand the freezing dry stress (Abdelwahed, 2015). On the basis of these findings, in future experiments, different lipids such as natural lipid theobroma oil and/or synthetic lipid tri-palmitic acid could be mixed with glyceryl trimyristate so as to increase the drug loading of LPM, and cryoprotectants would be tested for better reconstitution profiles.

The water solubility of loperamide is very limited (about 0.00086 mg/mL at 20°C). Tween 80 (Polysorbate 80, polyoxyethylene sorbitan monooleate) is a hydrophilic non-ionic surfactant widely used in emulsification and dispersion of substances in medicinal and food products (Strickley, 2004). Hence, in our dissolution experiment, 1% tween 80 was used for increasing the LPM solubility in the release medium to meet “sink condition.”

LD_50_ of LPM for oral administration in rats is 185 mg/kg. Oral doses (2.5–40 mg/kg) several times the antidiarrheal dose, unlike diphenoxylate, do not produce narcotic-like actions (Heel et al., 1978). In our preliminary experiments to screen the optimal loperamide dose for rats, four doses of loperamide (2.5, 10, 25, and 40 mg/kg) were tested. In case of the former two doses (2.5, 10 mg/kg), the drug plasma concentration was too low to be measured by HPLC, whereas the drug plasma concentrations after the latter two doses (25, 40 mg/kg) were well above lower limit of quantification. In order to avoid severe adverse effects on experimental animals, the lower dose of 25 mg/kg was chosen as optimal dosage for *in vivo* experiments.

## Conclusion

In this project, LPM-SLNs for oral delivery were successfully formulated by a solvent evaporation technique. The resultant LPM-SLNs formulations had relevant and homogeneous particle size with high encapsulation efficiency. It could be preserved and easily restored after lyophilization. *In vitro* experiments revealed a sustained release of the drug by LPM-SLNs, with little burst-release effect at pH 6.8. *In vivo* pharmacokinetic experiments indicated that the relative bioavailabilities of the LPM-SLN formulations were 227 and 153% higher than that of the LPM tablet in Wistar rats after oral administration. The SLNs could increase the gastrointestinal absorption of LPM. The nanostructured lipid vehicle provides a promising way to improve the oral bioavailability of water-insoluble drugs. The two different formulae were designed for different clinical usages. Based on these results, further investigations would be carried out to establish the exact mechanism underlying increased LPM oral absorption by this formulation.

## Author Contributions

LW and YY set up and carried out experiments, KS set up HPLC method and analyzed samples, JW executed data analysis, JM and WZ lead the research.

## Conflict of Interest Statement

The authors declare that the research was conducted in the absence of any commercial or financial relationships that could be construed as a potential conflict of interest.
